# Editorial: Quantitative modeling of psychopathology using passively collected data

**DOI:** 10.3389/fpsyg.2022.1126971

**Published:** 2023-01-24

**Authors:** Nicholas C. Jacobson, Burkhardt Funk, Saeed Abdullah

**Affiliations:** ^1^Center for Technology and Behavioral Health, Departments of Biomedical Data Science, Psychiatry, and Computer Science, Geisel School of Medicine, Dartmouth College, Hanover, NH, United States; ^2^Leuphana University Lüneburg, Lüneburg, Germany; ^3^College of Information Sciences and Technology, Penn State University, State College, PA, United States

**Keywords:** digital phenotyping, digital biomarker, mobile sensing, machine learning, passive sensing

Traditional mental health assessment collects data at a single point in time, which does not provide a comprehensive picture of an individual's mental health over an extended period. To address this issue, methods have been developed that involve repeatedly asking people to fill out surveys. However, these methods can be burdensome and as such, might not support granular assessment. Advances in technology have made it possible to collect a wide range of behavioral, social, and physiological data about an individual without requiring their active participation. This data can be collected from various sources, including smartphone and wearable sensors, electronic health records, social media data, and internet search data, with minimal burden to the individual. As a result, this data can provide a more comprehensive and nuanced understanding of an individual's mental health over time.

For instance, smartphone sensors can collect data on a person's social interactions, physical activity, and environment to gain insight into how these factors may affect their mental health. This data can be used to study the relationship between mental health measures and outcomes, develop predictive models, and test theoretical models of mental health. For instance, data from smartphone sensors could be used to explore the link between social isolation and changes in depression symptoms.

The goal of this Research Topic is to explore how passively collected data from sources like smartphones and wearable sensors can be used in combination with quantitative methods to improve our understanding and prediction of mental health. The manuscripts in this collection show how these data can be easily collected with minimal burden and can be used to gain new insights into mental health and to predict changes in mental health conditions.

Asgari et al. have developed a method for automatically detecting prosodic abnormalities in speech that are associated with autism. Using a machine learning model trained on a dataset of speech samples from individuals with autism and typically developing controls, they were able to accurately distinguish between the two groups. Their approach could be used as a new measure in autism treatment research and for early detection of the condition in preverbal infants or toddlers who are at risk.

Ponzo et al. have developed a new interoceptive task, the CARdiac Elevation Detection (CARED) task, which aims to improve upon existing methods. The task involves using a wearable device to record participants' heart rate and sending notifications to their mobile devices over a period of 4 weeks. Participants were asked to report on their heart rate and rate their confidence in their responses. The study found that the task was easy to administer and that participants had good insight into their interoceptive abilities.

Ding et al. developed a machine learning model that can predict changes in state anxiety levels with high temporal resolution. The model was trained on data collected from participants who were induced to experience state anxiety through exposure to aversive stimuli. The data included dimensional emotion ratings, electrocardiogram readings, and galvanic skin response measurements. The model was able to accurately predict self-reported state anxiety levels, providing a sensitive and fine-grained measure of state anxiety that could be useful for future studies of affective brain-computer interaction and anxiety modulation.

Liu and Shi present a novel hybrid feature selection and ensemble approach for detecting depression on social media. The method uses a combination of recursive elimination and extremely randomized trees to select the optimal subset of features for a stacking ensemble model. Their proposed method achieved an accuracy of 90.27% in identifying individuals with depression using online interaction data, resulting in a better performance than recent machine learning algorithms. This approach has potential applications for developing new methods to identify depression on social media.

Lin et al. evaluated the feasibility and effectiveness of using a machine learning-based smartphone app, the Ellipsis Health App, for detecting depression and anxiety in a senior population. The app uses semantic information from recorded speech to screen for these conditions. The study data shows a high completion rate of the app and a good performance in detecting depression and anxiety among seniors and various age ranges. Following the study findings, the authors suggest that the Ellipsis Health App is a promising tool for mental health screening in a senior population.

Maatoug et al. review the potential use of digital phenotyping in the diagnosis of mood disorders. Digital phenotyping involves the use of real-time data collected from digital sensors, wearable devices, and smartphones to determine the digital signature of a particular pathology. The study found that individuals with mood disorders often exhibit decreases in functional and biological parameters, such as decreased activities and walking, fewer calls and text messages, and lower body temperature and heart rate variability. These findings suggest that digital phenotyping could be a valuable addition to traditional clinical interviews in the diagnosis of mood disorders, providing objective data to supplement subjective symptoms.

The goal of this Research Topic is to examine how passively available data, such as data from smartphones and wearable sensors, can be used in combination with quantitative methods to improve our understanding and prediction of mental health. The manuscripts in this collection demonstrate how these data can be collected with minimal burden, used to enhance our knowledge of mental health, and/or used to predict mental health. They present a range of approaches, including using machine learning to detect prosodic abnormalities in speech, developing a wearable device for interoceptive research, and using multi-modal data to predict changes in anxiety levels. These approaches have the potential to improve the detection and monitoring of mental health conditions.

## Author contributions

All authors listed have made a substantial, direct, and intellectual contribution to the work and approved it for publication.

